# Identifying Adolescents at Risk for Depression: A Prediction Score Performance in Cohorts Based in 3 Different Continents

**DOI:** 10.1016/j.jaac.2019.12.004

**Published:** 2021-02

**Authors:** Thiago Botter-Maio Rocha, Helen L. Fisher, Arthur Caye, Luciana Anselmi, Louise Arseneault, Fernando C. Barros, Avshalom Caspi, Andrea Danese, Helen Gonçalves, Hona Lee Harrington, Renate Houts, Ana M.B. Menezes, Terrie E. Moffitt, Valeria Mondelli, Richie Poulton, Luis Augusto Rohde, Fernando Wehrmeister, Christian Kieling

**Affiliations:** a School of Medicine, Universidade Federal do Rio Grande do Sul, Brazil; b Division of Child & Adolescent Psychiatry, Hospital de Clínicas de Porto Alegre, Brazil; c National Institute of Developmental Psychiatry for Children and Adolescents, São Paulo, Brazil; d Social, Genetic & Developmental Psychiatry Centre, Institute of Psychiatry, Psychology & Neuroscience, King’s College London, United Kingdom; e National and Specialist CAMHS Clinic for Trauma, Anxiety, and Depression, South London and Maudsley NHS Foundation Trust, London, United Kingdom; f Postgraduate Program in Epidemiology, Universidade Federal de Pelotas, Pelotas, Brazil; g Duke University, Durham, North Carolina; h Institute of Psychiatry, Psychology & Neuroscience, King’s College London, United Kingdom; i National Institute for Health Research (NIHR) Maudsley Biomedical Research Centre, South London and Maudsley NHS Foundation Trust, London, United Kingdom; j Dunedin Multidisciplinary Health and Development Research Unit, University of Otago, Dunedin, New Zealand

**Keywords:** adolescent, cohort studies, depression, prognosis, risk assessment

## Abstract

**Objective:**

Prediction models have become frequent in the medical literature, but most published studies are conducted in a single setting. Heterogeneity between development and validation samples has been posited as a major obstacle for the generalization of models. We aimed to develop a multivariable prognostic model using sociodemographic variables easily obtainable from adolescents at age 15 to predict a depressive disorder diagnosis at age 18 and to evaluate its generalizability in 2 samples from diverse socioeconomic and cultural settings.

**Method:**

Data from the 1993 Pelotas Birth Cohort were used to develop the prediction model, and its generalizability was evaluated in 2 representative cohort studies: the Environmental Risk (E-Risk) Longitudinal Twin Study and the Dunedin Multidisciplinary Health and Development Study.

**Results:**

At age 15, 2,192 adolescents with no evidence of current or previous depression were included (44.6% male). The apparent C-statistic of the models derived in Pelotas ranged from 0.76 to 0.79, and the model obtained from a penalized logistic regression was selected for subsequent external evaluation. Major discrepancies between the samples were identified, impacting the external prognostic performance of the model (Dunedin and E-Risk C-statistics of 0.63 and 0.59, respectively). The implementation of recommended strategies to account for this heterogeneity among samples improved the model’s calibration in both samples.

**Conclusion:**

An adolescent depression risk score comprising easily obtainable predictors was developed with good prognostic performance in a Brazilian sample. Heterogeneity among settings was not trivial, but strategies to deal with sample diversity were identified as pivotal for providing better risk stratification across samples. Future efforts should focus on developing better methodological approaches for incorporating heterogeneity in prognostic research.

The field of prognostic research has seen a substantial rise in publications of prediction modeling studies in the last decade.^1^ This increase prompted significant advances in several medical specialties.^2^^,^^3^ However, most published prognostic models have been assessed in a single setting.^4^^,^^5^ Performance results obtained from model-development studies are frequently not achieved in validation trials when evaluated. This inconsistency can be explained either by an overoptimistic prognostic performance from an overfitted model or by significant discrepancies between development and validation samples.^6^

When assessing external validation across datasets, heterogeneity among prognostic studies is the norm rather than the exception.^7^ Differences in assessment strategies, frequency of outcome and/or studied factors, or availability of variables of interest could impose considerable difficulties for comparison purposes, impairing model generalizability. Current methodological guidelines recommend a set of careful development steps from derivation to external validation and ultimately use in clinical practice.^8^ In this process, understanding the similarities and differences between samples is essential,^9^ as guidelines suggest that a model with poor external performance should be updated before being discarded.^6^^,^^10^ This procedure integrates information obtained from new data to the developed model, potentially improving its prognostic ability.^4^^,^^11^ Even consolidated prediction models, such as the Framingham score for cardiovascular outcomes, face important drawbacks when applied in samples somewhat diverse from the original,^12^ demanding model adjustments to enhance generalizability to different settings.^4^^,^^6^

Up to now, the majority of psychiatric composite prognostic models studies have focused on model development, with very few being adequately validated in independent samples.^13^, ^14^, ^15^ In contrast to other areas of medicine, where hard outcomes are more easily defined, imprecise characterization of psychiatric outcomes imposes additional barriers for accurate prognostic model development and validation, as reliability of common mental disorders such as depression has been shown to be low.^16^ Substantial heterogeneity in clinical presentation and high rate of comorbidity produce additional obstacles for prediction of psychiatric disorders, as different assessment strategies influence the likelihood of endorsing a diagnosis.^17^

Prediction of psychosis, the most prolific and consolidated area in prognostic psychiatry, has greatly advanced at group level. However, it still faces challenges in prediction at the individual subject level.^18^ Prediction of major depressive disorder (MDD), the leading cause of mental health–related disease burden globally, is still in its infancy, relying mainly on single predictors for definition of at-risk people, with only a few studies combining risk factors.^19^ Following recently published standards for appropriate development and validation of psychiatric prediction models,^20^ using the most recent methodological recommendations^1^^,^^6^ and state-of-the-art statistical strategies,^21^^,^^22^ the present study aimed to derive and evaluate the generalizability of a psychiatric prediction model across samples from different sociocultural backgrounds.

Using data obtained from globally relevant longitudinal population-based cohorts, our first goal was to develop a multivariable prognostic model to evaluate the risk of developing a depressive episode by late adolescence in a Brazilian sample of adolescents with no evidence of previous depression, using a priori selected, easily obtainable sociodemographic variables collected directly from adolescents. Our second goal was to evaluate the impact of heterogeneity on its generalization to 2 diverse sociocultural contexts as well as to assess strategies to overcome these limitations.

## Method

### Samples and Participants

We derived our prediction model using data exclusively from the largest cohort available, the 1993 Pelotas Birth Cohort, a prospective study set in Brazil, and then evaluated the generalizability of findings in 2 diverse samples: the Environmental Risk (E-Risk) Longitudinal Twin Study, from the United Kingdom, and the Dunedin Multidisciplinary Health and Development Study, from New Zealand. Details about the 3 cohorts are reported elsewhere^23^, ^24^, ^25^ and in Supplement 1, available online. Briefly, in the Pelotas study, all 5,249 children born in the city of Pelotas in 1993 were enrolled in the study. The original goals of the 1993 Cohort were to evaluate trends in maternal and child health indicators to assess associations between early life variables and later outcomes. At the wave for ages 18–19 years old, the retention rate was 81.3% of the original sample. The Environmental Risk (E-Risk) Longitudinal Twin study tracks the development of a nationally representative birth cohort of 2,232 British twin children born in England and Wales in 1994–1995.^20^ The sample was constructed in 1999–2000, when 1,116 families with same-sex 5-year-old twins (93% of those eligible) participated in home-visit assessments. The Dunedin Study is a longitudinal investigation of health and behavior in a complete birth cohort. All study participants (N = 1,037; 91% of eligible births; 52% male) were born between April 1972 and March 1973 in Dunedin, New Zealand.

To be included in the final analysis, an evaluation for a depressive episode in late adolescence (18–19 years old) was required. Exclusionary criteria were applied, filtering out youths with intelligence quotient <70 and/or no signs of puberty by 15 years of age. Additionally, as our intention was to provide an alternative risk screening strategy beyond using previous depressive episodes or subthreshold depressive symptoms, participants with any suggestive evidence of a current or previous MDD diagnosis by the age of risk ascertainment were excluded from the final sample (see Table S1, available online). As the E-Risk sample was not evaluated at age 15, we selected the most comparable assessment wave, namely, age 12. Given the age difference at baseline between the E-Risk sample and the other samples, puberty was not considered an exclusionary criterion for this sample.

### Assessment and Definition of Predictor Variables

Selection of predictors was based on scientific literature review and authors’ clinical expertise,^26^ but constrained to their availability in the Pelotas dataset. As we aimed for real-world implementation, following a pragmatic approach,^27^ we included variables readily available, not too costly to obtain, and simple to evaluate.^20^^,^^22^ We adopted an a priori defined criterion to use only variables directly obtained from the adolescents in the Pelotas study at the age 15 assessment wave to mirror the reality in routine practice, selecting 11 variables related to inherent characteristics (biological sex, skin color), problematic behavior indicators (drug use, school failure, social isolation, fight involvement), and markers of household dysfunction (poor relationship with mother, poor relationship with father, poor relationship between parents, childhood maltreatment, ran away from home). For comparison purposes, the harmonization of selected variables among cohorts was performed a priori by consensus among investigators from each site. Further details on variables’ assessment strategies are provided in Table S1, available online.

### Assessment and Definition of the Outcome Variable

In each sample, the outcome of interest was a categorical diagnosis of depression in late adolescence. In the Pelotas cohort, trained psychologists interviewed the participants at ages 18–19 years in 2011–2012 with a structured interview for current MDD diagnosis using the Mini-International Neuropsychiatric Interview (MINI) based on *DSM-IV-TR* criteria, MDD section, assessing symptoms in the previous 2 weeks. For the E-Risk sample, MDD diagnosis in the previous 12 months was assessed using the Diagnostic Interview Schedule (DIS) at age 18 based on *DSM-IV* criteria in 2012–2014. In the Dunedin cohort, past-year MDD diagnosis was evaluated using the DIS at age 18 following *DSM-III-R* criteria in 1990–1991.

### Statistical Analysis

A detailed description of statistical procedures used can be found in Supplement 2, available online. In an effort to enhance the reproducibility of our model, we transparently described the process of model development and validation. Using data from the Pelotas cohort, we developed a baseline model using binary logistic regression (LR) analysis—the most common statistical strategy in prognostic research. As overfitting is a major reason for irreproducibility, we derived 6 new models from the same dataset introducing different strategies of model penalization—1 penalized LR model using penalized maximum likelihood estimation (PMLE) and 5 models with increasing degrees of penalization using the Elastic-Net machine learning algorithm.^21^ Comparing parameters of penalized models with our baseline model, we selected for validation the one with more balanced performance measures.

To evaluate the performance of the selected model in new observations, we first internally validated it using standard bootstrapping procedures to measure undue optimism in the model’s performance metrics, which happens when the model is evaluated directly in the derivation cohort (apparent performance). Second, we quantified the model’s prognostic performance in independent observations in 2 prospective cohorts from diverse contexts.

When assessing a given model’s prediction in independent samples, its performance may be influenced by differences between derivation and validation cohorts.^6^ Differences not only can be related to distribution of participant characteristics (case mix), but also can be true differences in predictor effects. To take this into account, we adopted a sequence of recommended approaches.^6^^,^^22^ We calculated a case mix–corrected and a refitted model for each sample, and the obtained metrics were used as performance parameters for each sample. Additionally, some of the originally selected variables were not available in all the cohorts, a likely situation in real-world model application. Instead of excluding these variables, we evaluated the amount of the original model’s information lost by this mismatch.^21^ Finally, we evaluated the impact of between-study heterogeneity by aggregating all cohorts into an overall sample to model cohort differences either in baseline risk or in predictor effects (see Supplement 3, available online).^28^

All statistical analyses were performed using R 3.4.4 software (R Foundation for Statistical Computing, Vienna, Austria). A complete-case analysis strategy was used, excluding participants with any missing data. A multiple imputation procedure using R package mice (R Foundation for Statistical Computing) was applied to assess missing data impact (see Table S2 and Figure S1, available online).

## Results

### Sample Characteristics

**Figure 1:**
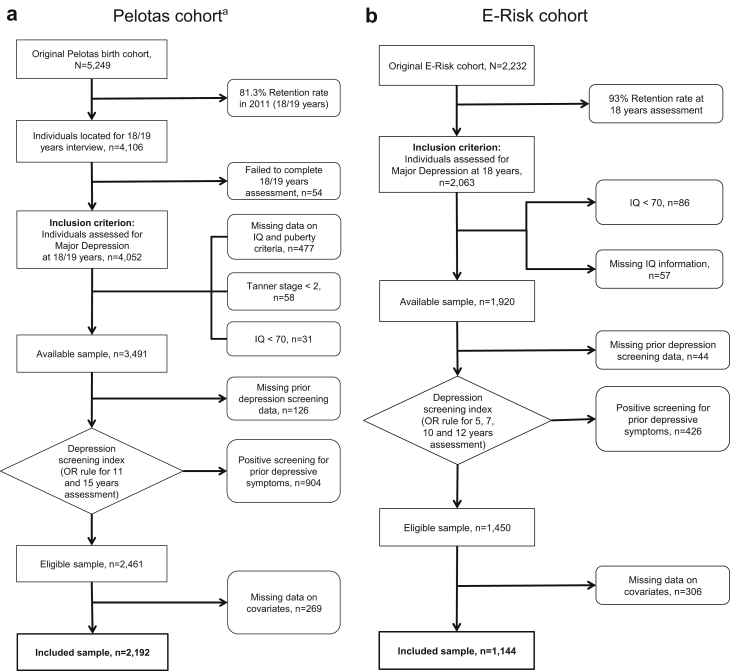

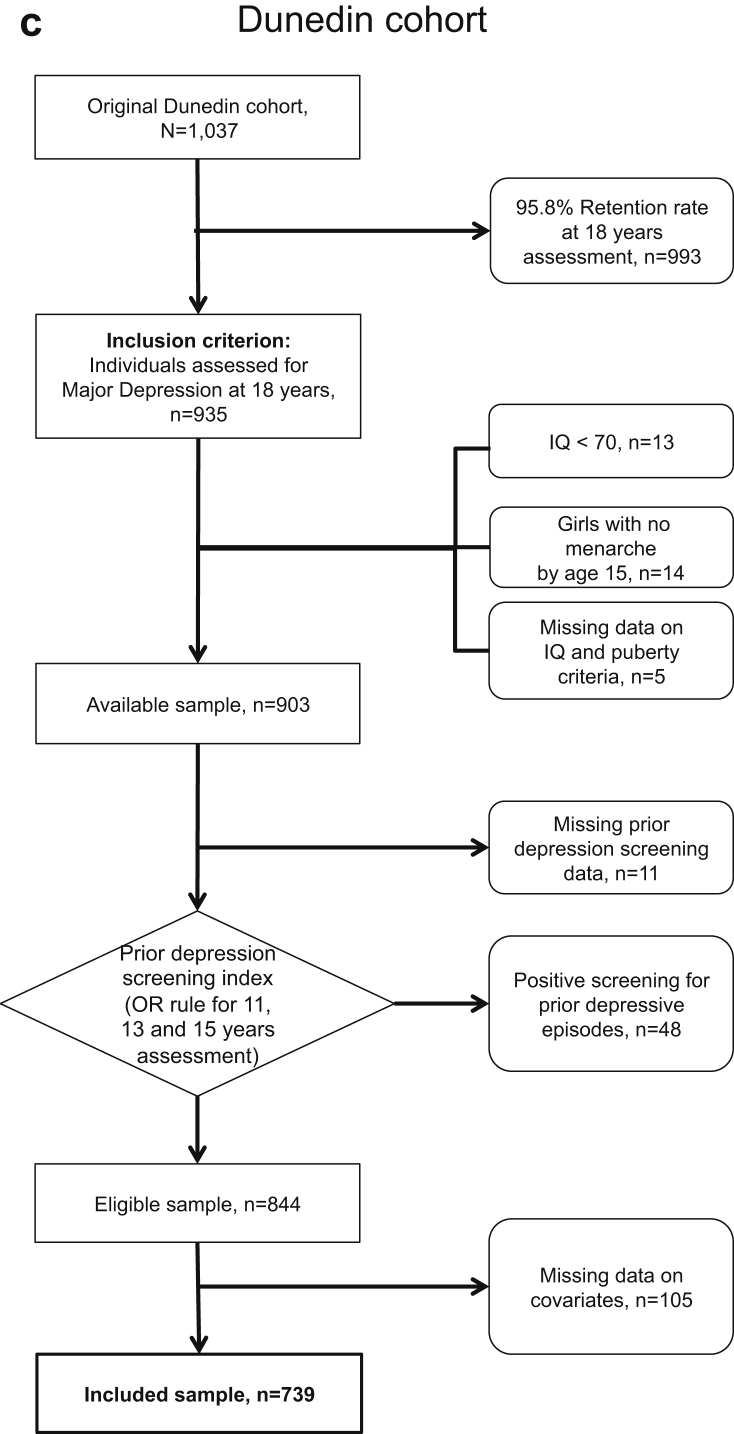
Flowcharts for Each Included Cohort Study ***Note:****(a) Pelotas cohort. (b) E-Risk cohort. (c) Dunedin cohort.* ^a^In the Pelotas dataset, 5 excluded participants had both Tanner < 2 and IQ <70.

A flowchart for each cohort is shown in Figure 1a–c. From the original sample size of 5,249 adolescents in the Pelotas cohort, 81.3% were retained up to the 18–19 years old assessment, and 2,192 were included for final analyses after applying exclusion criteria. For the E-Risk and Dunedin samples, from the 2,232 and 1,037 initially assessed adolescents, 1,144 (51.3%) and 739 (71.3%) were available for assessment after exclusion criteria were applied, respectively. Comparisons on key characteristics between retained and excluded samples for the Pelotas cohort are provided in Table S3, available online.

Table 1 presents descriptive variables for both depression outcome and selected predictors in each sample. Noteworthy disparities were identified regarding rates of school failure, social isolation, fight involvement, and running away. Additionally, family relationships were not assessed in the E-Risk Study. MDD prevalence in Pelotas, E-Risk, and Dunedin samples was 3.1%, 17.7%, and 16.8%, respectively. Differences in outcome prevalence among cohorts may have reflected differences in timeframe for outcome assessment (2 weeks versus 12 months).

**Table 1:** Sample Description for Each Cohort^a^

	Pelotas (Brazil)	E-Risk (United Kingdom)	Dunedin (New Zealand)
Included sample	2,192	1,144	739
Assessment age, years	15	12	15
Male sex	977 (44.6)^b^	520 (45.5)^b^	375 (50.7)^c^
White skin color	1,478 (67.4)^b^	1,040 (90.9)^c^	NA^e^
Childhood maltreatment			
None	1,539 (70.2)^b^	963 (84.2)^c^	489 (66.2)^d^
Probable	390 (17.8)	139 (12.2)	187 (25.3)
Severe	263 (12.0)	42 (3.7)	63 (8.5)
School failure	1,127 (51.4)^b^	212 (18.5)^c^	80 (10.8)^d^
Social isolation	231 (10.5)^b^	63 (5.5)^c^	70 (9.5)^b^
Fights	211 (9.6)^b^	130 (11.4)^b^	12 (1.6)^c^
Ran away from home	80 (3.6)^b^	9 (0.8)^c^	49 (6.6)^d^
Any drug use	1,367 (62.4)^b^	569 (49.7)^c^	592 (80.1)^d^
Relationship with mother		NA	
Great	1,417 (64.6)		
Very good	430 (19.6)		
Good	264 (12.0)		
Regular	68 (3.1)		
Bad	13 (0.6)		
Relationship with father		NA	22.0 ± 5.4^f^
Great	1,019 (46.5)		
Very good	434 (19.8)		
Good	370 (16.9)		
Regular	237 (10.8)		
Bad	132 (6.0)		
Relationship between parents		NA	
Great	886 (40.4)^b^		345 (46.7)^c^
Very good	421 (19.2)		278 (37.6)
Good	404 (18.4)		91 (12.3)
Regular	301 (13.7)		23 (3.1)
Bad	180 (8.2)		2 (0.3)
Depression prevalence	69 (3.1)^b^^,^^g^	202 (17.7)^c^^,^^h^	124 (16.8)^d^^,^^h^

Note: Results are shown as number of participants (percentage) for categorical variables and as mean ± SD for continuous variables for participants included in the final analyses. NA = Data not available in the cohort.

a See Table S1, available online, for assessment strategies applied to each cohort.

b–d Superscript letters b, c, and d denote column differences among the samples: different letters show significant differences and the same letters indicate nonsignificant differences from each other, assessed by χ^2^ test at .05 level. For variables with more than 2 categories, the superscript letters were placed in the first row of the variable and represent the assessment of the variable as a group, not per row.

e Skin color was not assessed in the cohort. Less than 7% of the cohort had any nonwhite ancestry.

f Parent Attachment Scale score (range, −6 to 28)—adolescent assessment about the relationship with both parents.

g Presence of symptoms reaching diagnostic criteria within a 2-week period before assessment.

h Presence of symptoms reaching diagnostic criteria within a 12-month period before assessment.

### Model Development and Validation

Performance measures showed better results for models using LR strategies compared with machine learning Elastic-Net approaches. In the Pelotas sample, discriminative capacity to parse between adolescents who later developed depression at age 18 and those who did not, assessed by the C-statistic, ranged from 0.76 to 0.79, indicating overall good discrimination, as shown in Table 2.

**Table 2:** Apparent Performance Parameters Obtained From the Models Derived From the Pelotas Dataset

	Model parameters						
	LR	PMLE^a^	Ridge^b^	.25^b^	.50^b^	.75^b^	LASSO^b^
*R*^*2*^	0.15	0.12	0.12	0.10	0.10	0.10	0.10
LR χ^2^^c^	81.90	66.17	63.30	54.40	54.32	54.71	54.10
Brier score^d^	2.88	2.93	2.93	2.95	2.95	2.95	2.95
C-statistic^e^	0.79	0.78	0.78	0.76	0.76	0.76	0.76
Calibration slope	1.00	1.26	1.35	1.47	1.42	1.38	1.39

Note: Higher results for *R*^*2*^, LR χ^2^, and C-statistic; lower results for Brier score; and results closer to 1 for calibration slope indicate better model performance. .25 = Elastic-Net with alpha = .25; .50 = Elastic-Net with α = .50; .75 = Elastic-Net with α = .75; Brier score = quadratic scoring rule that combines calibration and discrimination; C-statistic = concordance statistic, or area under the curve of the receiver operating characteristic; Calibration slope = measure of agreement between observed and predicted risk of the event (outcome) across the whole range of predicted values; LASSO = least absolute shrinkage and selection operator; LR = logistic regression; LR χ^2^ = likelihood ratio χ^2^; PMLE = penalized maximum likelihood estimation; *R*^*2*^ = Nagelkerke’s *R*^*2*^; Ridge = Ridge regression.

a The penalty factor used in the PMLE was empirically obtained from our data.

b For the Elastic-Net approach, we have a priori defined a grid of values for the hyperparameter α, ranging from 0 (full Ridge) to 1 (full LASSO), with increments of 0.25. For each α value, a 10-fold cross-validation was used to select the penalty coefficient (λ) that minimized the mean squared prediction error, which was then used for shrinkage of coefficients and/or variable selection. See Table S4, available online, for model’s coefficients.

c All LR χ^2^*p* values < .001.

d Multiplied by 10^2^.

e The C-statistic ranges from 0.5 for noninformative models to 1.0 for perfect models.

Predictably, the baseline model showed the best combination of performance metrics. Among penalized models, the PMLE model demonstrated better performance compared with all Elastic-Net models. As nonpenalized models face a greater risk of overfitting, we proceeded to the next step with both LR models for comparison. We internally validated each using bootstrapping evaluation with 1,000 iterations. As expected, measurement of optimism—difference between apparent and bias-corrected performance metrics—was lower for the PMLE model compared with the LR model (ΔC-statistic: 0.067 versus 0.098; Δslope: −0.004 versus 0.548; Δ*R*^*2*^: 0.034 versus 0.149), suggesting lower overfitting and higher probability of reliable results when applied to independent samples. Additionally, as shown in Figure S2a–b, the PMLE model was also more calibrated, with a 60% reduction in mean square error compared with the LR model. Therefore, the PMLE model was selected as the Pelotas final model, with a C-statistic of 0.78 (bootstrap-corrected 95% CI: 0.73–0.82).

Using the most common external validation strategy, the linear predictor derived from the selected Pelotas model (Table S4, available online) was applied to the other samples. There was an expected decrease in the performance metrics in both independent cohorts (E-Risk: C-statistic 0.59 [bootstrap-corrected 95% CI: 0.55–0.63]; Dunedin: C-statistic 0.63 [bootstrap-corrected 95% CI: 0.59–0.67]). The performance results for each step of the validation process are presented in Table 3.

**Table 3:** Comparative Results for Each Step of Model Performance in the 3 Cohorts

Performance parameter	Description	Pelotas		E-risk			Dunedin		
		Apparent validation	Internal validation	External validation	Case mix–corrected model^a^	Refitted model^b^	External validation	Case mix–corrected model^a^	Refitted model^b^
C-statistic	Concordance statistic, equal to area under the curve of receiver operating characteristic in binary endpoints	0.78	0.71	0.59	0.66	0.62	0.63	0.68	0.67
Calibration-in-the-large	Overall measure of calibration, compares mean observed with mean predicted in validation dataset	0.00	0.02	2.37	0.02	0.00	2.26	−0.06	0.00
Calibration slope	Measure of agreement between observed and predicted risk of event (outcome) across whole range of predicted values	1.26	1.00	0.58	0.99	1.20	0.77	0.98	1.24
*R*^*2*^	Measure of overall goodness-of-fit of model	0.12	0.06	0.03	0.04	0.05	0.05	0.05	0.09
Brier score	Quadratic scoring rule that combines calibration and discrimination	0.03	0.03	0.17	0.02	0.14	0.16	0.02	0.13
Emax	Maximum absolute error in predicted probabilities	0.19	0.03	0.29	0.01	0.09	0.38	0.01	0.11
**Available information for assessment of model performance**		100%		86.9%			93.1%		

Note: Higher results for C-statistic and *R*^*2*^, lower results for Brier score and Emax, results closer to 0 for calibration-in-the-large, and results closer to 1 for calibration slope indicate better model performance.

a Reference values indicating the model’s performance under the assumption that Pelotas model’s coefficients are fully correct for the validation setting, simulating similar case mix between samples.^22^

b Reference values indicating the model’s performance after refitting predictors’ coefficients that would be optimal for the validation sample.^22^ (See Supplement 2, available online, for further details.)

### Model Updating

As variables from both independent datasets did not perfectly pair with the set selected from the Pelotas study, we calculated the amount of information lost owing to this mismatch.^21^ In the E-Risk dataset, 13.1% of original model information was unavailable, mainly from the household dysfunction indicators. In the Dunedin dataset, this percentage was lower, at around 6.9%.

Considering the relevant heterogeneity among cohorts, we evaluated whether the integration of information from the external cohorts could produce improvement in model performance, in line with current methodological recommendations.^4^ As differences in outcome prevalence were not trivial, we updated the Pelotas model by correcting its intercept for each cohort. In both validation samples, the updated model produced better calibration, reducing all measures of calibration error (Supplement 2 and Figure S3a–d, available online).

### Exploratory Analyses

**Figure 2:**
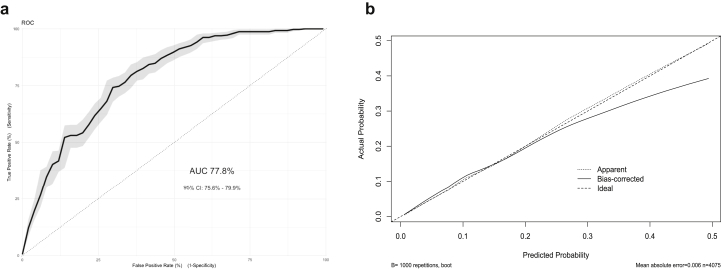
Performance Measures of the Aggregated Sample Model ***Note:****(a) The area under the curve (AUC) of the receiver operating characteristic (ROC) curve and the bootstrapped 95% CI (indicated by gray shading) of the C-statistic, and (b) calibration plot after internal validation using 1,000 iterations bootstrapping. Apparent and bias-corrected results were plotted as a nonparametric calibration curve, estimated over a sequence of predicted values versus observed values using a smoothing technique.*

**Figure 3:**
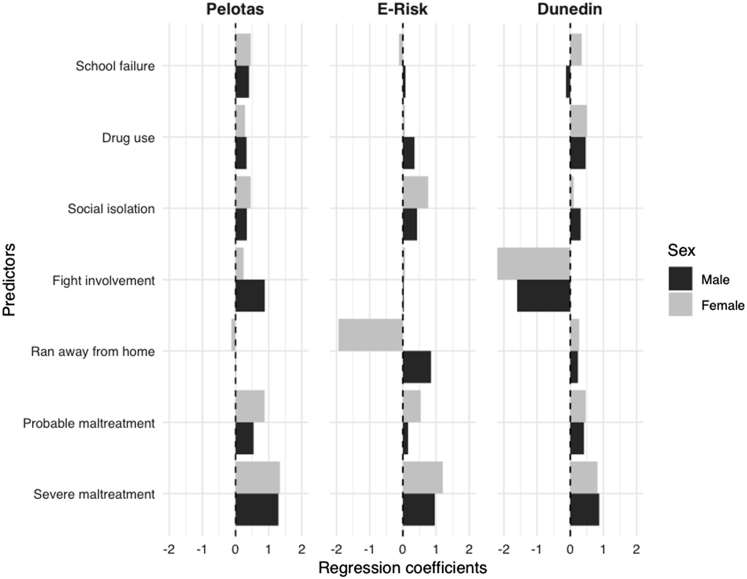
Prognostic Contribution of Each Included Variable to the Aggregated Sample Prediction Model of Adolescent Depression ***Note:****Comparison of the prognostic contribution of each included variable in each cohort to the aggregated sample prediction model of adolescent depression, stratified by sex for Brazil, United Kingdom, and New Zealand cohorts. Predictors’ β coefficients from penalized logistic regression are shown as bars in the x-axis. Positive values represent greater risk and negative values represent lower risk of the outcome. The results shown are derived from values presented in*Table S5*, available online. Some of the variables previously included in the Pelotas model were excluded for comparability among datasets.*

The merger of all 3 cohorts into an aggregated sample to assess between-cohort heterogeneity increased the total number of participants to 4,075, of which 395 (9.7%) demonstrated a positive outcome. Given that most of the participants were from the Pelotas cohort (53.8%), the C-statistic was also 0.78 (bootstrap-corrected 95% CI: 0.75–0.80), but showed lower overfitting after internal validation using bootstrapping (Figure 2a–b). Inclusion of each cohort’s main effects and their interaction terms with all predictors into a PMLE model suggested that not only disparities in case mix, as shown in Table 1, but also between-cohort differences in predictor effects might have influenced external validation results, particularly considering the difference in the ran-away and fight involvement variables (Figure 3).

## Discussion

Following current standards for psychiatric prognostic research,^20^ our study proposes a multivariable model developed in a Brazilian cohort to predict among adolescents with no evidence of previous depression the risk of developing a depressive episode in late adolescence. Our model showed beyond chance results of discrimination and calibration, with metrics comparable to established prognostic models from other areas of medicine,^3^^,^^29^ and could be viewed as a promising aid to adolescent depression risk stratification.^30^

Evaluation in independent samples is deemed essential for generalization of findings. Disparities among samples are frequently seen as major obstacles for model validation, replication, and generalizability. However, as the Transparent Reporting of a multivariable prediction model for Individual Prognosis Or Diagnosis (TRIPOD) statement emphasizes, the term validation can be misleading, recommending that an external validation should quantify the model’s prognostic performance in a new sample, not simply classifying it as a positive or negative validation.^4^^,^^31^^,^^32^ This broader validation approach not only promotes the assessment of the model’s performance in the new sample but also facilitates understanding of why the results differ.

For this study, we assessed the validation performance of the model developed in our Brazilian sample in 2 population-based longitudinal cohorts from 2 different continents. The development of a model in 1 middle-income country and its external validation in samples representing diverse sociocultural and economic contexts, using different assessment strategies for data collection at different time periods among them, may help evaluate if and where its results can be generalized. Our results suggest that, albeit adaptations should be applied to the original model to enhance external clinical utility, the original prognostic model could be applied in multiple other contexts despite major differences in assessment strategies, socioeconomic characteristics, and cultural influences. Given such profound differences, it was expected that the developed model could not be easily transported to new settings.^9^ Even though lower in degree, our model kept a valid and beyond chance prognostic capacity in parsing future risk of depression among the adolescents in the independent cohorts, especially when heterogeneity among samples was accounted for (Supplement 3 and Figure S3a–d, available online).

Early identification of people at higher risk for psychiatric disorders could potentially lessen the massive burden imposed by these conditions. Positive family history of depression and the presence of subthreshold depressive symptoms have been the most commonly used criteria for identifying at-risk children and adolescents.^33^ Although these strategies have been replicated, reliance on single predictors restricts their prognostic contribution, not accounting for a wider range of risk. Additionally, from a pragmatic perspective, the requirement of trained staff for proper evaluation of such predictors limits their potential implementation, given that access to treatment has been systematically highlighted as a major barrier for child and adolescent mental health care.^34^

Our study has several strengths. We developed a prognostic model for MDD according to most recent guidelines in prognostic research and transparent reporting^6^^,^^20^ using modern, state-of-the-art statistical strategies^21^^,^^22^ with broad external validation assessment. Comprising only 11 predictors, all easily obtainable, quick to assess, and collected directly from the adolescent, with no need for highly specialized training, external informants, or laboratory analyses, our results could be seen as promising if further replicated. Additionally, consistent with the evidence-based pragmatic psychiatry initiative,^27^ we opted to prioritize simplicity over accuracy, selecting predictors that could be more easily and broadly implemented, enhancing probability of future clinical use and patient acceptance.

Significant limitations of our study also need to be considered. Having based the development of our prognostic model on the Pelotas cohort, an ongoing study not primarily focused on mental health, availability of variables of interest was limited to those previously collected, precluding the use of some potentially relevant factors. MDD diagnosis was assessed at the age 18–19 years wave by evaluating symptoms in the 2 weeks before the interview, limiting comparability to other epidemiological cohort studies as well as reducing the prevalence of the outcome of interest. Consequently, the number of outcome events per selected variable was lower in the Pelotas sample (events per variable = 6.27), increasing the risk of overfitting.^20^, ^21^, ^22^ Strategies such as machine learning regularization methods, with shrinkage and selection of predictors as well as measurement of performance optimism, were implemented to constrain the impact of this limitation. The proposed model is also not necessarily prognostic of earlier or later onsets of depression.^35^ Furthermore, as we were analyzing participants at higher risk of MDD diagnosis, we could not discard the chance that all self-report assessments were biased by this risk. Additionally, as our goal was to provide a risk stratification tool that could be supplementary to current strategies of risk evaluation, we opted to exclude participants with any evidence of previous or current depressive episodes because the occurrence of a depressive episode already heightens the risk of subsequent depression. This strategy resulted in a significant number of exclusions that could have biased our findings; therefore, we compared the covariates between included and excluded samples (Table S3, available online), with anticipated differences between them, and performed sensitivity analyses (see Table S6 and Figure S4, available online) in which similar performance results were identified.

The differences in predictors’ availability and assessment strategies among cohorts are another relevant shortcoming, which could have influenced results obtained in the external validations. The unavailability of assessment data at age 15 in the E-Risk sample could have impacted the comparability among the samples, as puberty is a well-known risk contributor for depression,^36^ and could therefore have contributed to the performance result of the model in that sample. A priori harmonization of variables and measurement of information lost as a result of mismatching variables were applied to minimize the effect of these limitations. Also, we were constrained to variables assessed in each cohort study, which precluded important predictors being included in our model, and the included variables could be carrying prognostic information from uncollected predictors, which could have contributed to discrepancies in predictor effects shown in Figure 3. Finally, in the present study, we could not evaluate the potential impact of the developed model on clinical decision making.^20^

Exploratory analyses suggested that information generated by our model increased prognostic ability above and beyond established risk factors, such as subsyndromal symptoms and a positive family history of depression (Supplement 4 and Table S7, available online). At the same time, the risk score was also associated, to a lesser degree, with other diagnostic outcomes (C-statistic range: 0.64–0.70) (Table S8, available online). In line with the current literature on the early detection of psychopathology in youth,^37^ we believe that a transdiagnostic approach could be considered, despite its limitations,^38^ as specificity of psychiatric prognostic models is likely to be low and as less specific preventive interventions could promote meaningful changes in psychiatric burden, either from individual or public health perspectives.^9^^,^^39^

In conclusion, we present the development of a prognostic model for MDD among Brazilian adolescents, externally evaluated in 2 samples from diverse sociocultural contexts using different strategies for data collection than the original cohort. Heterogeneity among studies was high and possibly accounted for major discrepancies in prognostic performance, probably related not only to different case mix but also to weight of coefficients.^6^ Future studies should pursue methodological strategies for embracing heterogeneity among samples, instead of avoiding it, thus producing results that are more likely to be translated into clinical practice across a range of contexts.
