# Supplementary material for: Identifying Adolescents at Risk for Depression: A Prediction Score Performance in Cohorts Based in 3 Different Continents
Source: J Am Acad Child Adolesc Psychiatry. 2021 Feb;60(2):262–73. doi: 10.1016/j.jaac.2019.12.004 (PMC8215370; doi:10.1016/j.jaac.2019.12.004)
Supplement: Supplemental Material [file mmc1.docx]

**Supplement 1**

**Samples description**

In 1993, all 5,249 children born live in the city of Pelotas were enrolled in the study. At the wave for ages 18-19 years old, the retention rate was 81.3% of the original sample. Further information on the cohort design can be found elsewhere.^1,2^ Pelotas currently has around 300,000 inhabitants and is located at the extreme South of Brazil, near the Uruguayan border, a region with higher degrees of European genetic contributions when compared to other parts of Brazil. Its main economic activities are rice production, commerce and education. At the time of the beginning of the study, the infant mortality rate was 21 deaths per thousand births. Participants have been followed up at several points in time during infancy, childhood, adolescence and young adulthood. The original goals of the 1993 Cohort were to evaluate trends in maternal and child health indicators, through a comparison with results of the 1982 study, the first of a series of birth cohort studies performed in the city, to assess associations between early life variables and later outcomes, with particular emphasis on the detection of critical windows; and to improve data quality, using the lessons learned from the 1982 study.

The Environmental Risk (E-Risk) Longitudinal Twin study tracks the development of a nationally-representative birth cohort of 2,232 British twin children born in England and Wales in 1994-1995.^3^ Families were recruited to represent the UK population of families with newborns in the 1990s, based on residential location throughout England and Wales and mothers’ age. E-Risk families are representative of UK households across the spectrum of neighborhood-level deprivation.^4^ The sample comprised 56% monozygotic and 44% dizygotic twin pairs, and sex was evenly distributed within zygosity (49% male). Follow-up home-visits were conducted when children were aged 7, 10, 12, and 18 years (participation rates were 98%, 96%, 96%, and 93%, respectively). The Joint South London and Maudsley and the Institute of Psychiatry Research Ethics Committee approved each phase of the study. Parents gave informed consent and twins gave assent between 5-12 years and then informed consent at age 18.

The Dunedin Study represents the full range of socioeconomic status on NZ’s South Island and matches the NZ National Health and Nutrition Survey on key health indicators (e.g., BMI, smoking, GP visits).^5^ The cohort is primarily white; fewer than 7% self-identify as having non-Caucasian ancestry, matching the South Island. Assessments were carried out at birth and ages 3, 5, 7, 9, 11, 13, 15, 18, 21, 26, 32, and, most recently, 38 years, when 95% of the 1,007 study members still alive took part. At each assessment, each study member is brought to the research unit for a full day of interviews and examinations. These data are supplemented by questionnaires completed by persons who know the study members well and by official record searches.

**Table S1. Comparative table of variables’ definitions and assessment strategies among the included studies**

| **Variable**  **(reference category)^a^** | **Pelotas** | **E-Risk** | **Dunedin** |
| --- | --- | --- | --- |
| Previous depression screening | Any evidence (“OR” rule) of depressive symptoms, assessed by borderline score in emotional SDQ subscale for self and parent assessment at age 11 and for parent assessment at age 15 | Any evidence (“OR” rule) of depressive symptoms, assessed at ages 5, 7 and 10 by a depression subscale derived from a combination of mother and teacher CBCL for emotional problems, using the 93^th^ percentile as cutpoint, and at age 12 by self-reported CDI scores (with a clinical cut-off >= 20) | Any positive depressive episode assessment using the DISC at ages 11, 13 or 15 |
| Puberty | Second stage of Tanner classification at age 15 | Not applied in the sample (see main text) | Menarche for girls at age 15^b^ |
| Sex  (Male) | Self reported sex | Self reported sex | Self reported sex |
| Skin color  (White) | Self assigned skin color | Self assigned skin color | Self assigned ancestry |
| Childhood maltreatment (None) | Responses to seven dichotomous questions regarding lifetime psychological, physical and sexual abuse and/or neglect at age 15;^6^ zero positive answers=none, 1 positive=probable, 2 or more=severe; inserted as a categorical variable into the model | Prospectively obtained variable for sexual/physical abuse up to age 12 based on mother reports, researcher observations, and social services referral information, coded as none, probable, or definite; inserted as a categorical variable into the model | Prospectively obtained variable for childhood maltreatment up to age 12 based on mother reports, researcher observations, social services referral information, and retrospective self-reports, coded none/probable/severe; inserted as a categorical variable into the model |
| School failure (No) | In Brazil, if a child fails to achieve a predetermined score at the end of the school year, the child is retained by the school to repeat the same school year; positive answers to the dichotomous question: “Have you ever been retained in school?” was classified as “failing at school”=1; otherwise=0 | Evaluation of sample’s distribution of English/Math performance at age 12, considering those below the 20^th^ percentile as “failing at school”=1; otherwise=0 | Those who left school at age 15 with no qualifications were classified as “failing at school”=1; otherwise=0 |
| Social isolation (0) | Responses to the question: “Do you normally meet up with friends to chat, play or do other things? If YES, how many days in a given week?” were dichotomized as follows: responses “no” were classified as “1” and “yes” were classified as “0” | Combination of CBCL and TRF items on social isolation was pooled into a 3 strata categorical social isolation variable (low, moderate and high social isolation)^7^, and then reclassified into a dichotomized variable: high social isolation=1 and low/moderate=0 | Those below the 10^th^ percentile of the sample’s distribution in the Peer Attachment Scale were classified as “socially isolated”=1; those above it were classified as “socially isolated”=0 |
| Fights  (No) | Responses to the dichotomous question: “In the last year, have you ever gotten into a physical fight that someone got hurt?”; positive answers were classified as “1”, and negative as “0” | Combination (“AND” rule) of two dichotomous questions: “Do you sometimes hit someone when you are having an argument?” and “Do you sometimes start fights with people?”; positive answers to both questions were classified as “1”; otherwise, “0” | Response to the question: “In the past year, how many times have you fought in the street or other public place?”; regrouped into a dichotomized variable: once or more=“1” and never=“0” |
| Ran away  (No) | Responses to the dichotomous question: “Have you ever run away from home?”; positive answers were classified as “1”, and negative as “0” | Responses to the dichotomous question: “Have you run away from home and stayed away for the night?”; positive answers were classified as “1”, and negative as “0” | Question about running away overnight; regrouped into a dichotomized variable: once or more=“1” and never=“0” |
| Drug use  (No) | Dichotomous variable combining responses to dichotomous questions about any lifetime use of alcohol, tobacco, cannabis, cocaine and inhalants; any positive answer=“1”; otherwise=“0” | Dichotomous variable combining responses to dichotomous questions about any lifetime use of alcohol, tobacco, cannabis, pills and inhalants; any positive answer=“1”; otherwise=“0” | Dichotomous variable combining responses to dichotomous questions about any lifetime use of alcohol, tobacco, cannabis, inhalants and other illegal drug; any positive answer=“1”; otherwise=“0” |
| Relationship with mother  (Great) | Question: “How do you rate your relationship with your mother?”; choices of answers: great=1, very good=2, good=3, regular=4, bad=5; inserted as a categorical variable into the model | No match | Continuous variable obtained from responses to the Parent Attachment Scale^c^ |
| Relationship with father  (Great) | Question: “How do you rate your relationship with your father?”; choices of answers: great=1, very good=2, good=3, regular=4, bad=5; inserted as a categorical variable into the model | No match |  |
| Relationship between parents  (Great) | Question: “How do you rate the relationship between your father and your mother?”; choices of answers: great=1, very good=2, good=3, regular=4, bad=5; inserted as a categorical variable into the model | No match | Parents arguing frequency; choice of answers: never=1, one or two times=2, sometimes=3, often=4, all the time=5; inserted into the model as a categorical variable |
| Depression diagnosis at age 18 (No) | Evaluation of major depressive episode diagnosis with DSM-IV-TR criteria in the previous two weeks^d^ | Evaluation of major depressive episode diagnosis with DSM-IV criteria in the previous 12 months | Past-year major depressive episode using DIS at age 18 following the DSM-III-R criteria |

^a^For variables included as predictors.

^b^No puberty assessment available for boys in the cohort.

^c^See Methods S2 for details.

^d^ All psychiatric diagnoses were assessed by trained psychologists using an instrument derived from the Mini International Neuropsychiatric Interview.^8^

CBCL: Child Behavior Checklist; CDI: Children’s Depression Inventory; DIS: Diagnostic Interview Schedule; DISC: Diagnostic Interview Schedule for Children; DSM: Diagnostic and Statistical Manual of Mental Disorders; SDQ: Strengths and Difficulties Questionnaires; TRF: Teacher’s Report Form.

For comparison purposes, the harmonization among the cohorts of the selected variables was performed a priori, when consensus was reached with each cohort’s primary investigator before datasets sharing and assessment.

**Table S2. Coefficients comparison for the selected Pelotas model in complete-case analyses and after multiple imputation strategy**

|  | **Complete-case analyses** | **Multiple imputation** |
| --- | --- | --- |
| Intercept | -4.642 | -4.687 |
| Sex | 0.325 | 0.270 |
| Skin color | -0.030 | -0.096 |
| School failure | 0.290 | 0.442 |
| Drug use | 0.121 | 0.156 |
| Social isolation | 0.127 | 0.147 |
| Fights involvement | 0.580 | 0.588 |
| Ran away from home | -0.017 | 0.028 |
| Probable maltreatment | 0.422 | 0.362 |
| Severe maltreatment | 0.652 | 0.755 |
| Relat. w/mother=2 | 0.054 | 0.133 |
| mother=3 | 0.161 | 0.209 |
| mother=4 | -0.026 | 0.009 |
| mother=5 | 0.006 | 0.024 |
| Relat. w/father=2 | -0.010 | 0.005 |
| father=3 | 0.297 | 0.359 |
| father=4 | 0.181 | 0.205 |
| father=5 | 0.237 | 0.320 |
| Relat. bw parents=2 | -0.004 | -0.017 |
| parents=3 | 0.251 | 0.186 |
| parents=4 | 0.163 | 0.194 |
| parents=5 | 0.037 | 0.130 |
| Sex*Skin color | 0.170 | 0.110 |
| Sex*School failure | 0.114 | 0.097 |
| Sex*Drug use | 0.108 | 0.130 |
| Sex*Social isolation | 0.269 | 0.222 |
| Sex*Fights | -0.395 | -0.376 |
| Sex*Ran away | -0.101 | -0.211 |
| Sex*Probable maltreatment | 0.369 | 0.411 |
| Sex*Severe maltreatment | 0.382 | 0.313 |
| Sex*Relat. w/mother=2 | -0.219 | -0.206 |
| Sex*mother=3 | 0.060 | 0.029 |
| Sex*mother=4 | -0.203 | -0.239 |
| Sex*mother=5 | -1.293 | -1.386 |
| Sex*Relat. w/father=2 | 0.143 | 0.154 |
| Sex*father=3 | -0.279 | -0.201 |
| Sex*father=4 | -0.103 | -0.150 |
| Sex*father=5 | 0.537 | 0.416 |
| Sex*Relat. bw parents=2 | 0.011 | 0.079 |
| Sex*parents=3 | -0.035 | -0.094 |
| Sex*parents=4 | 0.158 | 0.118 |
| Sex*parents=5 | 0.075 | 0.149 |

Complete-case analyses: Coefficients obtained from a penalized logistic regression model using penalized maximum likelihood estimation (PMLE) after list-wise exclusion due to missing data on covariates. Multiple imputation: Coefficients obtained after multiple imputation using 10 imputation datasets and 10 iterations using the method of chained equations (R package *mice*). Frequency of missing data for each parameter (from highest to lowest): Relationship with father: 93; Relationship between parents: 81; Childhood maltreatment: 59; Ran away: 46; Relationship with mother: 43; Drug use: 29; Fights: 19. Relat. w/mother: adolescent’s relationship with his(her) mother; Relat. w/father: adolescent’s relationship with his(her) father; Relat. bw parents: relationship between parents (for all three, reference category 1="great"). Interaction term is shown as “variable”*”variable”.

**Figure S1. Predictive performance of the Pelotas model after multiple imputation**

Calibration indicates the agreement between predicted probabilities and observed frequencies. A calibration plot is a graphical assessment of calibration, where the calculated predicted probability is shown on the x-axis, and observed frequency of the outcome on the y-axis. The flexible calibration line shows the result of a smoothing technique (Loess algorithm) used to estimate the observed probabilities of the binary outcome in relation to the predicted probabilities.

The grey shaded area indicates the 95% confidence interval around the estimated calibration line. The logistic calibration curve shows the result of the estimation of the observed proportions when a logistic model is used for the outcome as a function of the linear predictor of the developed model. The 45-degree line (labelled ‘Ideal’) has slope=1 and indicates perfect calibration. PMLE: Penalized maximum likelihood estimation; LR: Logistic regression. C (ROC): C-statistic; R2: Nagelkerke’s R^2^; D: Discrimination index; Brier: Brier score; ECI: Estimated Calibration Index; Intercept: Regression intercept of linear predictor; Slope: Regression slope of linear predictor.

**Table S3. Pelotas sample characteristics for individuals included and excluded from the final analyses**

|  | **Included** | **Excluded** | **Total**^a^ |
| --- | --- | --- | --- |
| Male sex | 977 (44.6%)^b^ | 1006 (54.1%)^c^ | 1983 (48.9%) |
| White skin color | 1478 (67.4%)^b^ | 1024 (59.9%)^c^ | 2502 (64.1%) |
| Childhood maltreatment |  |  |  |
| No | 1539 (70.2%)^b^ | 914 (62.5%)^c^ | 2453 (67.1%) |
| Probable | 390 (17.8%)^b^ | 274 (18.7%)^b^ | 664 (18.2%) |
| Severe | 263 (12.0%)^b^ | 275 (18.8%)^c^ | 538 (14.7%) |
| School failure | 1127 (51.4%)^b^ | 1301 (76.1%)^c^ | 2428 (62.2%) |
| Social isolation | 231 (10.5%)^b^ | 266 (15.5%)^c^ | 497 (12.7%) |
| Any drug use | 1367 (62.4%)^b^ | 992 (61.5%)^b^ | 2359 (62.0%) |
| Fights | 211 (9.6%)^b^ | 242 (14.9%)^c^ | 453 (11.9%) |
| Ran away | 80 (3.7%)^b^ | 108 (6.8%)^c^ | 188 (5.0%) |
| Relationship with mother |  |  |  |
| Great | 1417 (64.6%)^b^ | 853 (53.4%)^c^ | 2270 (59.9%) |
| Very good | 430 (19.6%)^b^ | 342 (21.4%)^b^ | 772 (20.4%) |
| Good | 264 (12.0%)^b^ | 257 (16.1%)^c^ | 521 (13.8%) |
| Regular | 68 (3.1%)^b^ | 119 (7.5%)^c^ | 187 (4.9%) |
| Bad | 13 (0.6%)^b^ | 25 (1.6%)^c^ | 38 (1.0%) |
| Relationship with father |  |  |  |
| Great | 1019 (46.5%)^b^ | 606 (40.3%)^c^ | 1625 (44.0%) |
| Very good | 434 (19.8%)^b^ | 269 (17.9%)^b^ | 703 (19.0%) |
| Good | 370 (16.9%)^b^ | 290 (19.3%)^b^ | 660 (17.9%) |
| Regular | 237 (10.8%)^b^ | 218 (14.5%)^c^ | 455 (12.3%) |
| Bad | 132 (6.0%)^b^ | 120 (8.0%)^c^ | 252 (6.8%) |
| Relationship between parents |  |  |  |
| Great | 886 (40.4)%^b^ | 520 (34.3%)^c^ | 1406 (37.9%) |
| Very good | 421 (19.2%)^b^ | 288 (19.0%)^b^ | 709 (19.1%) |
| Good | 404 (18.4%)^b^ | 285 (18.8%)^b^ | 689 (18.6%) |
| Regular | 301 (13.7%)^b^ | 243 (16.0%)^c^ | 544 (14.7%) |
| Bad | 180 (8.2%)^b^ | 180 (11.9%)^c^ | 360 (9.7%) |
| Depressive episode | 69 (3.1%)^b^ | 93 (5.0%)^c^ | 162 (4.0%) |
| Total | 2,192 | 1,860 | 4,052 |

^a^ Total number of individuals meeting our inclusion criteria. The sum of included and excluded samples differs from the total number shown in some rows due to 457 individuals with missing data for exclusionary criteria. Categorical variables presented as percentages (according to column). Results derived from a chi-square (χ^2^) test. Superscript letters “b” and “c” denote column differences between included and excluded samples: different letters show significant and equal letters indicate non-significant differences from each other at a 0.05 level.

**Table S4. Variables’ regression coefficients for each developed model from the Pelotas dataset**

|  | **LR** | **PMLE** | **Ridge** | **0.25** | **0.50** | **0.75** | **LASSO** |
| --- | --- | --- | --- | --- | --- | --- | --- |
| Intercept | -5.879 | -4.642 | -4.313 | -3.963 | -3.948 | -3.978 | -3.961 |
| Sex | 1.569 | 0.325 | 0.137 | 0.047 | 0.002 | . | . |
| Skin color | -0.284 | -0.030 | 0.004 | . | . | . | . |
| School failure | 0.863 | 0.290 | 0.152 | 0.034 | . | . | . |
| Drug use | 0.160 | 0.121 | 0.081 | . | . | . | . |
| Social isolation | 0.673 | 0.127 | 0.125 | . | . | . | . |
| Fights involvement | 1.582 | 0.580 | 0.491 | 0.336 | 0.350 | 0.364 | 0.330 |
| Ran away from home | -0.539 | -0.017 | 0.001 | . | . | . | . |
| Probable maltreatment | 0.148 | 0.422 | 0.210 | 0.063 | . | . | . |
| Severe maltreatment | 1.107 | 0.652 | 0.509 | 0.612 | 0.767 | 0.890 | 0.955 |
| Relat. w/mother=2 | 0.894 | 0.054 | 0.025 | . | . | . | . |
| mother=3 | 0.045 | 0.161 | 0.197 | 0.232 | 0.234 | 0.233 | 0.216 |
| mother=4 | -6.321 | -0.026 | -0.185 | . | . | . | . |
| mother=5 | -6.430 | 0.006 | -0.596 | . | . | . | . |
| Relat. w/father=2 | -1.103 | -0.010 | -0.091 | . | . | . | . |
| father=3 | 1.242 | 0.297 | 0.177 | . | . | . | . |
| father=4 | 0.811 | 0.181 | 0.107 | . | . | . | . |
| father=5 | 1.258 | 0.237 | 0.302 | 0.107 | . | . | . |
| Relat. bw parents=2 | -0.356 | -0.004 | -0.065 | . | . | . | . |
| parents=3 | 0.365 | 0.251 | 0.177 | 0.007 | . | . | . |
| parents=4 | -0.010 | 0.163 | 0.127 | . | . | . | . |
| parents=5 | -0.897 | 0.037 | -0.036 | . | . | . | . |
| Sex*Skin color | 0.488 | 0.170 | 0.144 | 0.045 | 0.021 | 0.012 | . |
| Sex*School failure | -0.463 | 0.114 | 0.169 | 0.201 | 0.257 | 0.279 | 0.283 |
| Sex*Drug use | 0.093 | 0.108 | 0.124 | 0.075 | 0.058 | 0.051 | 0.038 |
| Sex*Social isolation | -0.189 | 0.269 | 0.236 | 0.169 | 0.166 | 0.177 | 0.147 |
| Sex*Fights | -1.615 | -0.395 | -0.250 | . | . | . | . |
| Sex*Ran away | 0.386 | -0.101 | -0.061 | . | . | . | . |
| Sex*Probable maltreatment | 0.966 | 0.369 | 0.399 | 0.505 | 0.651 | 0.710 | 0.714 |
| Sex*Severe maltreatment | 0.240 | 0.382 | 0.379 | 0.287 | 0.188 | 0.092 | 0.025 |
| Sex*Relat. w/mother=2 | -1.337 | -0.219 | -0.126 | . | . | . | . |
| Sex*mother=3 | 0.068 | 0.060 | 0.076 | . | . | . | . |
| Sex*mother=4 | 5.839 | -0.203 | -0.013 | . | . | . | . |
| Sex*mother=5 | -1.361 | -1.293 | -0.716 | -0.110 | -0.039 | -0.056 | . |
| Sex*Relat. w/father=2 | 1.260 | 0.143 | 0.150 | . | . | . | . |
| Sex*father=3 | -1.499 | -0.279 | -0.164 | . | . | . | . |
| Sex*father=4 | -0.949 | -0.103 | -0.013 | . | . | . | . |
| Sex*father=5 | -0.492 | 0.537 | 0.459 | 0.565 | 0.691 | 0.713 | 0.715 |
| Sex*Relat. bw parents=2 | 0.304 | 0.011 | 0.039 | . | . | . | . |
| Sex*parents=3 | -0.162 | -0.035 | 0.009 | . | . | . | . |
| Sex*parents=4 | 0.347 | 0.158 | 0.161 | 0.111 | 0.089 | 0.084 | 0.066 |
| Sex*parents=5 | 0.890 | 0.075 | 0.129 | . | . | . | . |

LR: Logistic regression model; PMLE: penalized maximum likelihood estimation logistic regression; 0.25: Elastic-Net with alpha=0.25; 0.50: Elastic-Net with alpha=0.50; 0.75: Elastic-Net with alpha=0.75; Relat. w/mother: adolescent’s relationship with his(her) mother; Relat. w/father: adolescent’s relationship with his(her) father; Relat. bw parents: relationship between parents (for all three, reference category 1="Great"). Interaction term is shown as “variable”*”variable”.

**Figure S2a-b. Calibration plots after internal validation using 1,000 iterations bootstrapping for: a) the logistic regression (LR), and b) the Penalized maximum likelihood estimation (PMLE) LR model.**

Apparent and bias-corrected results were plotted as a nonparametric calibration curve, estimated over a sequence of predicted values vs. observed values using a smoothing technique.

**Supplement 2**

**Complementary description of statistical analysis**

From a predictive modeling perspective, the use of the full-model, the one that contains all pre-specified terms, will usually be the one that predicts most accurately in new data (model generalization or validation).^9^ However, the maintenance of all pre-selected variables in the final model can sometimes produce overfitting. Overfitting can be described as an overly optimistic predictive estimate of accuracy, biasing the developed model’s generalization. Several statistical strategies have been developed to deal with overfitting, where a penalization factor is included into the model for shrinkage of coefficients and/or variable selection.^9-11^ Shrinkage (or Penalization) strategies such as Penalized Maximum Likelihood Estimation (PMLE), Ridge regression, LASSO (Least Absolute Shrinkage and Selection Operator), and Elastic-Net penalization have been used in the literature for reducing overfitting and variance, optimizing prediction on new data.^9^ The Elastic-Net machine learning technique blends both Ridge and LASSO penalization approaches,^12^ having the advantage of combining shrinkage of parameters with selection of meaningful variables, excluding non-contributing factors without the risks of p value-based variable selection techniques.^9,13^

Following current recommendations of transparent reporting,^13,14^ we describe here a stepwise description of the strategy for statistical analysis:

**Step one:**

For the Pelotas sample, no transformation or data handling was performed, keeping the variables as they were originally collected. All predictors were entered as categorical variables into the models. Given that biological sex is a consolidated risk factor for depression,^15^ we have included into the models interaction terms of sex with all other selected variables, as well as their main effects. The penalty factor used in the PMLE was empirically obtained from our data. For the Elastic-Net approach, we have *a priori* defined a grid of values for the hyperparameter alpha, ranging from 0 (full Ridge) to 1 (full LASSO), with increments of 0.25. For each alpha value, a 10-fold cross-validation was used to select the penalty coefficient (lambda) that minimized the mean squared prediction error, which was then used for shrinkage of coefficients and/or variable selection. The Elastic-Net models with alphas > 0 had some variables excluded from final analyses, as their contributions after penalization were negligible (see Table S4).

**Step two:**

Discrimination and calibration metrics, paired with calibration plots, are the commonest performance measures for model assessment.^13,16^ The discriminative capacity of the model is defined as the ability to differentiate low- from high-risk individuals. For binary outcomes, discrimination can be quantified by the AUC, also named concordance statistic (C-statistic). Calibration refers to the agreement of predicted and observed outcomes, and can be investigated by calibration plots using flexible calibration curves or categorizations of predicted risk. Additionally to models’ discrimination and calibration measures, results of explained variation (R^2^), model’s goodness-of-fit (Likelihood Ratio χ^2^), predictive accuracy (Brier score), the discrimination index (D), and the maximum error in predicted probabilities (Emax) were also calculated.^9,16^

**Step three:**

A predictive model generally performs better in the sample used to develop the model, namely apparent performance, compared to other samples. This performance measures’ discrepancy is called model optimism. Assessment of optimism is crucial for estimating the chance of a model’s reproducibility and implementation. We have submitted the models to 1,000 resampling iterations bootstrapping, the most recommended internal validation process,^9,17^ to get bias(overfitting)-corrected estimates. Then, this bias-corrected model was plotted as a nonparametric calibration curve, estimated over a sequence of predicted values vs. observed values using a smoothing technique.

Considering the non-matching variables in the E-Risk and Dunedin samples, there was a need to rebuild the Pelotas model for each of the external validation samples, recalculating the linear predictor considering only the variables available for each comparison. Also, strategies for enhancing comparability between datasets were implemented. Since there was only a combined parent relationship assessment variable in the Dunedin sample, with no adequate matching to the separated evaluation of adolescent relationship with each parent, we have decided to transform the Pelotas variables for better comparison to the Dunedin dataset. After transforming both variables (relationship with mother and relationship with father) into continuous variables, we have derived the arithmetical mean of both variables (summed them and divided by two). As the Dunedin study used the Parent Attachment Scale, a different evaluation strategy, with a larger range of results, with higher results suggesting a better relationship, we have also Z-scored both the obtained arithmetical mean and the Parent Attachment Scale results for better comparability. Given that the Pelotas coding of parent relationship assessment variables have an opposite direction (higher value, worse relationship) to the Dunedin variable, we have multiplied its coefficient value by -1. As mentioned in the main text, there were no family relationship assessment available in the E-Risk dataset for comparison.

**Model update:**

Current methodological guidelines recommend the identification of calibration-in-the-large problems should have priority when evaluating external validation, since miscalibration can cause systematically wrong decision making. Model updating (or adjustment) intends to make the average predicted probability equal to the observed overall event rate by fitting a new logistic regression model in the validation sample using the new intercept as the only free parameter, with the original linear predictor (obtained from the development sample) as an offset variable.

The adjustment of Pelotas model by correcting its intercept for each cohort, resulted in an improvement of calibration measures. In the E-Risk dataset, this adjustment reduced the Brier score by 14.3% (from 0.17 to 0.14) and the Emax value by 11.2% (from 0.29 to 0.26), keeping the remaining metrics unchanged. A similar reduction was also achieved in Dunedin: 14.6% and 66.6% for Brier and Emax scores, respectively.

**Figure S3a-d. Calibration plots for standard and adjusted (updated) external validation assessment in each sample.**

Calibration indicates the agreement between predicted probabilities and observed frequencies. A calibration plot is a graphical assessment of calibration, where the calculated predicted probability is shown on the x-axis, and observed frequency of the outcome on the y-axis. The flexible calibration line shows the result of a smoothing technique (Loess algorithm) used to estimate the observed probabilities of the binary outcome in relation to the predicted probabilities.

The grey shaded area indicates the 95% confidence interval around the estimated calibration line. The logistic calibration curve shows the result of the estimation of the observed proportions when a logistic model is used for the outcome as a function of the linear predictor of the developed model. The 45-degree line (labelled ‘Ideal’) has slope=1 and indicates perfect calibration. The distribution of predicted probabilities is shown at the bottom of the graphs.

Calibration in the large: compares the mean of all predicted risks with the mean observed risk. Calibration slope: measure of agreement between observed and predicted risk of the outcome across the whole range of predicted values. C-statistic: Concordance statistic, equal to the area under the curve of the receiver operating characteristic (AUC-ROC) in binary endpoints.

**Supplement 3**

**Model validation analyses**

For evaluation of relatedness of the development and validation samples, we have followed current recommendations for assessment of disparities in case mix.^18,19^ Firstly, we compared the distribution of predictors of the validated model, as well as the outcome of interest, among samples (Table 1). Secondly, we have evaluated the dispersion of the model’s predicted risk in the development and validation samples. Assessing the spread and mean value of the linear predictor (lp) of the derived model in the development and validation samples, in the Pelotas sample, the mean value of lp was -3.678, with a spread, measured by the standard deviation (SD), of 0.685. In the E-Risk sample, the mean lp was -4.012 and SD was 0.562. For the Dunedin sample, the mean lp was -3.983, with a SD of 0.601. Finally, we assessed predictors’ coefficients for the variables available in all cohorts after merging all datasets into an aggregated sample, rebuilding the PMLE model after including each cohort’s main-effects and their interaction terms with all predictors (cohort*predictor), as can be seen in Table S5 and Figure 3.

Additionally, following recommended statistical strategies,^20^ we quantified the impact of differences in case-mix on the model’s validation performance. For this analysis, we assume that the regression coefficients for assessed predictors and the model intercept are fully correct for the validation setting. Simulating the outcome from the observed case-mix in the development sample, assuming the prediction model is correct for the new sample, differences in performance between the development and validation assessments suggest real differences in the regression coefficients’ weights.

We have also calculated the performance obtained by refitting the model in the validation data, estimating coefficients that would be optimal for the validation data. This approach provides an upper bound for model performance if the coefficients from the development setting were exactly equal to those in the validation setting. As this upper bound is a result of both case-mix and the effects of predictors in the validation setting, differences in performance results could be related to both.^20^

**Table S5. Exploratory analysis after merging all datasets from Pelotas, E-Risk and Dunedin cohorts for variables available in all cohorts^a^**

|  | Coefficient | SE |
| --- | --- | --- |
| Intercept | -5.027 | 0.355 |
| Sex | 0.857 | 0.301 |
| School failure | 0.407 | 0.295 |
| Drug use | 0.332 | 0.261 |
| Social isolation | 0.341 | 0.470 |
| Fights involvement | 0.882 | 0.394 |
| Ran away from home | 0.005 | 0.653 |
| Probable Child. Mt | 0.544 | 0.340 |
| Severe Child. Mt | 1.293 | 0.403 |
| E-Risk cohort | 2.749 | 0.354 |
| Dunedin cohort | 2.345 | 0.404 |
| Sex*School failure | 0.053 | 0.273 |
| Sex*Drug use | -0.047 | 0.230 |
| Sex*Social isolation | 0.117 | 0.460 |
| Sex*Fights | -0.640 | 0.514 |
| Sex*Ran away | -0.131 | 0.662 |
| Sex*Probable Child. Mt | 0.334 | 0.330 |
| Sex*Severe Child. Mt | 0.048 | 0.403 |
| Sex*E-Risk | -0.026 | 0.289 |
| Sex*Dunedin | -0.094 | 0.355 |
| School failure*E-Risk | -0.329 | 0.351 |
| School failure*Dunedin | -0.535 | 0.496 |
| Drug use*E-Risk | 0.018 | 0.274 |
| Drug use*Dunedin | 0.133 | 0.298 |
| Social isolation*E-Risk | 0.086 | 0.567 |
| Social isolation*Dunedin | -0.028 | 0.566 |
| Fights*E-Risk | -0.854 | 0.445 |
| Fights*Dunedin | -2.483 | 1.490 |
| Ran away*E-Risk | 0.844 | 1.026 |
| Ran away*Dunedin | 0.227 | 0.750 |
| Probable Child. Mt*E-Risk | -0.387 | 0.419 |
| Severe Child. Mt*E-Risk | -0.330 | 0.558 |
| Probable Child. Mt*Dunedin | -0.130 | 0.396 |
| Severe Child. Mt*Dunedin | -0.419 | 0.559 |
| Sex*School failure*E-risk | -0.250 | 0.412 |
| Sex*School failure*Dunedin | 0.421 | 0.623 |
| Sex*Drug use*E-Risk | -0.245 | 0.301 |
| Sex*Drug use*Dunedin | 0.085 | 0.345 |
| Sex*Social isolation*E-Risk | 0.219 | 0.674 |
| Sex*Social isolation*Dunedin | -0.328 | 0.761 |
| Sex*Fights*E-Risk | 0.675 | 0.665 |
| Sex*Fights*Dunedin | -1.069 | 3.249 |
| Sex*Ran away*E-Risk | -2.660 | 3.462 |
| Sex*Ran away*Dunedin | 0.169 | 0.835 |
| Sex*Probable Child. Mt*E-Risk | 0.049 | 0.479 |
| Sex*Severe Child. Mt*E-Risk | 0.193 | 0.746 |
| Sex*Probable Child. Mt*Dunedin | -0.281 | 0.459 |
| Sex*Severe Child. Mt*Dunedin | -0.099 | 0.653 |

^a^ Due to the requirement of availability of variables in all datasets for this exploratory analysis, Pelotas model’s coefficients had their values recalculated, as some previously included variables were excluded for comparability.

Interaction term is shown as “variable”*”variable” or “variable”*”variable”*”variable”.

Mt: maltreatment. SE: standard error.

To calculate the coefficients shown in Figure 3 of the manuscript, the above coefficient values were summed considering variables sex and cohort, having male sex and Pelotas cohort as references. For instance, for estimation of the coefficient corresponding to the “Fights involvement” variable for females in the Dunedin cohort, this variable’s main-effect (0.882) was summed to the interaction term sex*Fights (-0.640), to the interaction term Fights*Dunedin (-2.483), and to the interaction term sex*fights*Dunedin (-1.069), resulting a coefficient value of -3.309.

**Table S6. Sensitivity analysis assessing the impact of the exclusion criteria on the Pelotas model**

|  | **Included sample**  (n=2,192) | **Available sample**  (n=3,290) |
| --- | --- | --- |
| Intercept | -4.642 | -4.564 |
| Sex | 0.325 | 0.416 |
| Skin color | -0.030 | -0.107 |
| School failure | 0.290 | 0.205 |
| Drug use | 0.121 | 0.069 |
| Social isolation | 0.127 | 0.031 |
| Fights involvement | 0.580 | 0.480 |
| Ran away from home | -0.017 | -0.119 |
| Probable maltreatment | 0.422 | 0.420 |
| Severe maltreatment | 0.652 | 0.582 |
| Relat. w/mother=2 | 0.054 | -0.006 |
| mother=3 | 0.161 | 0.188 |
| mother=4 | -0.026 | 0.029 |
| mother=5 | 0.006 | 0.041 |
| Relat. w/father=2 | -0.010 | 0.040 |
| father=3 | 0.297 | 0.332 |
| father=4 | 0.181 | 0.122 |
| father=5 | 0.237 | 0.172 |
| Relat. bw parents=2 | -0.004 | 0.001 |
| parents=3 | 0.251 | 0.241 |
| parents=4 | 0.163 | 0.245 |
| parents=5 | 0.037 | 0.014 |
| Sex*Skin color | 0.170 | 0.169 |
| Sex*School failure | 0.114 | 0.098 |
| Sex*Drug use | 0.108 | 0.025 |
| Sex*Social isolation | 0.269 | 0.463 |
| Sex*Fights | -0.395 | -0.649 |
| Sex*Ran away | -0.101 | -0.168 |
| Sex*Probable maltreatment | 0.369 | 0.332 |
| Sex*Severe maltreatment | 0.382 | 0.504 |
| Sex*Relat. w/mother=2 | -0.219 | -0.246 |
| Sex*mother=3 | 0.060 | 0.210 |
| Sex*mother=4 | -0.203 | -0.023 |
| Sex*mother=5 | -1.293 | 0.598 |
| Sex*Relat. w/father=2 | 0.143 | 0.167 |
| Sex*father=3 | -0.279 | -0.139 |
| Sex*father=4 | -0.103 | 0.068 |
| Sex*father=5 | 0.537 | 0.589 |
| Sex*Relat. bw parents=2 | 0.011 | 0.248 |
| Sex*parents=3 | -0.035 | 0.044 |
| Sex*parents=4 | 0.158 | -0.044 |
| Sex*parents=5 | 0.075 | 0.388 |

Included sample: Coefficients obtained from the penalized logistic regression model using penalized maximum likelihood estimation (PMLE) in Pelotas dataset for those included in final analyses, after exclusionary criteria. Available sample: Coefficients obtained from the penalized logistic regression model using PMLE in Pelotas dataset for all individuals meeting our inclusion criterion with no missing data on covariates (complete-case analysis). Relat. w/mother: adolescent’s relationship with his(her) mother; Relat. w/father: adolescent’s relationship with his(her) father; Relat. bw parents: relationship between parents (for all three, reference category 1="Great"). Interaction term is shown as “variable”*”variable”.

**Figure S4. Predictive performance of the Pelotas model using all available sample**

Calibration indicates the agreement between predicted probabilities and observed frequencies. A calibration plot is a graphical assessment of calibration, where the calculated predicted probability is shown on the x-axis, and observed frequency of the outcome on the y-axis. The flexible calibration line shows the result of a smoothing technique (Loess algorithm) used to estimate the observed probabilities of the binary outcome in relation to the predicted probabilities.

The grey shaded area indicates the 95% confidence interval around the estimated calibration line. The logistic calibration curve shows the result of the estimation of the observed proportions when a logistic model is used for the outcome as a function of the linear predictor of the developed model. The 45-degree line (labelled ‘Ideal’) has slope=1 and indicates perfect calibration. The distribution of predicted probabilities is shown at the bottom of the graphs. PMLE: Penalized maximum likelihood estimation; LR: Logistic regression. C (ROC): C-statistic; R2: Nagelkerke’s R^2^; D: Discrimination index; Brier: Brier score; ECI: Estimated Calibration Index; Intercept: Regression intercept of linear predictor; Slope: Regression slope of linear predictor.

**Supplement 4**

**Exploratory analyses**

In our study, supplementary strategies were implemented to further evaluate our model’s properties in terms of predictive performance above and beyond currently established risk factors for depression; outcome specificity; and concentration of risk metrics.

We first assessed the relationship between our proposed model and two currently used strategies to identify adolescents at risk for developing depression: subsyndromal symptoms and family history of depression. Given our *a priori* decision not to rely on depressive symptoms as predictors, and only use variables directly obtained from the adolescent, we tested whether the inclusion of sub-threshold depressive symptoms and family history of depression, established risk factors for MDD, could provide additional predictive information to the model. Sub-threshold depressive symptoms were evaluated using the emotional sub-scale of the Strengths and Difficulties Questionnaire (SDQ) – parent-report at the 15-years assessment (no self-report measure was available for this age); and family history of depression was assessed using the maternal Self-Report Questionnaire (SRQ), only available for the 11-years assessment in Pelotas cohort study.

Net reclassification indices or improvement (NRI) have recently become popular statistics for measuring the prediction increment of new variables.^21^ Measures of reclassification quantify the extent to which individuals are more appropriately classified into risk categories using a new model versus an old model. Individuals are placed into predefined risk categories based on their predicted absolute risk of experiencing the outcome (event) according to each model. Reclassification can be quantified using the NRI, that is the sum of 2 proportions: the proportion of events that move up through the risk categories upon using the new model; and the proportion of nonevents that move down through the risk categories upon using the new model. Current literature suggest the reporting of the “event NRI” (or NRI+) and “nonevent NRI” (NRI-) along with an overall NRI.

The impact of the inclusion of new variables into the Pelotas model for prediction of a depressive episode is shown in Table S7. Overall, the inclusion of these predictors failed to produce substantial improvements to the Pelotas model. The contribution of consolidated risk factors such as family history of depression (SRQ, either continuous or above the recommended threshold^22^) or sub-threshold depressive symptoms (emotional sub-scale of the SDQ – parent report) either alone or in combination, as shown in the first major row of the Table S7, produced only a small impact in all reclassification measures. These results suggest that the Pelotas model can provide predictive information to such an extent that the inclusion of further established risk factors could not produce meaningful classification improvement.

Additionally, we further assessed whether the developed risk score would behave differently according to either a positive depression screening or family history of depression status. By independently introducing each risk factor’s main-effects and interaction terms with the Pelotas model linear predictor as covariates of the linear predictor itself for the depression outcome in Pelotas dataset, our results did not suggest a modification of predictive performance related to any of the factors’ status (data available upon request).^23^

Conversely, we also evaluated whether the Pelotas model could provide an aid to decision-making strategies commonly used in current clinical settings. Using as baseline comparators three reduced models with consolidated risk factors for depressive disorder, namely biological sex, positive family history of depression and evidence of sub-threshold depressive symptoms, we aimed to assess if the use of Pelotas model could result in improvement above and beyond the information carried by these three models. As shown in the second major row, the added value of the Pelotas model to the baseline models was relevant in all measures. Evidence of greater and significant impact for the NRI- when compared to the NRI+ suggests its potential usefulness in reducing false-positive findings.

As a second step, we also evaluated the prediction performance of the Pelotas model linear predictor for all evaluated diagnoses at the 18-19 years’ assessment in the Pelotas cohort, as can be seen in Table S8. The composite score was able to predict, to a lesser extent in comparison to the prediction of MDD, all other diagnostic categories assessed in the 1993 Pelotas Cohort.

Finally, as a third exploratory approach, we assessed the usefulness of the set of selected factors capacity itself in parsing high and low risk individuals across settings, after refitting the models in each dataset, using complementary metrics potentially useful in clinical decision-making (concentration of risk (CR) – the proportion of cases in the highest tenth of risk;^24^ and high/low tenths ratio (HLTR) – the ratio between the proportion of cases in the highest and in the lowest tenths of the predicted risk range). In this conceptual validation, the Pelotas model’s parsing capacity was high, with a CR of 31.9% and an incalculable HLTR (31.9/0), as its lowest tenth included only non-depressed participants. Similarly, for the E-Risk model, the CR was 18.3%, with a HLTR of 3.7, and the Dunedin model obtained a CR of 21.8%, with a HLTR of 3.9.

**Table S7. Net Reclassification Improvement (NRI) analyses**

|  |  | **NRI** | **NRI+** | **NRI-** | **ΔC-statistic** |
| --- | --- | --- | --- | --- | --- |
| Impact of predictor inclusion into Pelotas model | **Continuous SRQ** | 0.041  (-0.004 – 0.096) | 0.043  (0.000 – 0.100) | -0.002  (-0.011 – 0.006) | 0.001 |
|  | **Categorical SRQ** | 0.005  (-0.050 – 0.062) | 0.000  (-0.056 – 0.057) | 0.005  (-0.003 – 0.013) | -0.001 |
|  | **Sub-threshold symptoms** | 0.037  (-0.038 – 0.121) | 0.029  (-0.050 – 0.112) | 0.008  (-0.004 – 0.021) | 0.019 |
|  | **Continuous SRQ +**  **Sub-threshold symptoms** | 0.036  (-0.040 – 0.119) | 0.029  (-0.044 – 0.110) | 0.007  (-0.006 – 0.021) | 0.021 |
| Impact of Pelotas model inclusion into reduced models | **Reduced model 1** | 0.222  (0.085 – 0.348) | 0.043  (-0.092 – 0.171) | 0.179  (0.155 – 0.202) | 0.135 |
|  | **Reduced model 2** | 0.200  (0.068 – 0.328) | -0.058  (-0.188 – 0.069) | 0.258  (0.234 – 0.279) | 0.145 |
|  | **Reduced model 3** | 0.199  (0.051 – 0.342) | 0.116  (-0.031 – 0.258) | 0.083  (0.059 – 0.107) | 0.142 |

Classification improvement according to the NRI (95% CI) at event rate.^21^ NRI+: NRI for events (positive outcomes); NRI-: NRI for non-events (negative outcomes); ΔC-statistic: Change in C-statistic. SRQ: Self-Report Questionnaire; Categorical SRQ: SRQ>=8; Sub-threshold symptoms: According to emotional sub-scale of the Strengths and Difficulties Questionnaire (SDQ) – parent-report at the 15 years assessment in Pelotas.

Reduced model 1: sex and continuous SRQ; Reduced model 2: sex and categorical SRQ; Reduced model 3: sex, continuous SRQ and sub-threshold depressive symptoms.

Positive values indicate improvement and negative values indicate worsening in classification.

**Table S8. Outcome specificity assessment of Pelotas model in the development sample**

|  | **R^2^** | **C-Statistic** | **Calibration intercept** | **Calibration slope** | **Brier score** | **Emax** |
| --- | --- | --- | --- | --- | --- | --- |
| ADHD | 0.05 | 0.70 | -0.36 | 0.82 | 0.02 | 0.26 |
| BD | 0.03 | 0.66 | -0.87 | 0.71 | 0.01 | 0.49 |
| GAD | 0.06 | 0.69 | 0.78 | 0.80 | 0.06 | 0.07 |
| MDD | 0.12 | 0.78 | 0.00 | 1.25 | 0.03 | 0.19 |
| SAD | 0.03 | 0.64 | 0.55 | 0.58 | 0.05 | 0.31 |

Prediction performance of the Pelotas model linear predictor for all evaluated diagnoses at the 18/19 years assessment in the Pelotas cohort. All psychiatric diagnoses were assessed by trained psychologists using an instrument derived from the Mini International Neuropsychiatric Interview.^8^

R^2^: Nagelkerke’s R^2^; C-statistic: Concordance statistic, or area under the curve of the receiver operating characteristic (AUC-ROC); Calibration intercept: relates to calibration-in-the-large, which compares the mean of all predicted risks with the mean observed risk; Calibration slope: measure of agreement between observed and predicted risk of the event (outcome) across the whole range of predicted values; Brier score: Quadratic scoring rule that combines calibration and discrimination; Emax: Maximum absolute error in predicted probabilities. ADHD: Attention-deficit/hyperactivity disorder; BD: Bipolar disorder; GAD: Generalized anxiety disorder; MDD: Major depressive disorder; SAD: Social anxiety disorder.

**References**

1. Victora CG, Araújo CL, Menezes AM, et al. Methodological aspects of the 1993 Pelotas (Brazil) Birth Cohort Study. *Rev Saude Publica.* 2006;40(1):39-46.

2. Victora CG, Hallal PC, Araújo CL, Menezes AM, Wells JC, Barros FC. Cohort profile: the 1993 Pelotas (Brazil) birth cohort study. *Int J Epidemiol.* 2008;37(4):704-709.

3. Moffitt TE, E-Risk Study Team. Teen-aged mothers in contemporary Britain. *J Child Psychol Psychiatry.* 2002;43(6):727-742.

4. Odgers CL, Caspi A, Russell MA, Sampson RJ, Arseneault L, Moffitt TE. Supportive parenting mediates neighborhood socioeconomic disparities in children’s antisocial behavior from ages 5 to 12. *Dev Psychopathol*. 2012;24(3):705-721.

5. Poulton R, Moffitt TE, Silva PA. The Dunedin Multidisciplinary Health and Development Study: overview of the first 40 years, with an eye to the future. *Soc Psychiatry Psychiatr Epidemiol.* 2015;50(5):679-693.

6. Rocha TB, Hutz MH, Salatino-Oliveira A, et al. Gene-environment interaction in youth depression: replication of the 5-HTTLPR moderation in a diverse setting. *Am J Psychiatry.* 2015;172(10):978-985.

7. Matthews T, Danese A, Wertz J, et al. Social isolation and mental health at primary and secondary school entry: a longitudinal cohort study. *J Am Acad Child Adolesc Psychiatry.* 2015;54(3):225-232.

8. Amorim P. Mini International Neuropsychiatric Interview (MINI): validação de entrevista breve para diagnóstico de transtornos mentais. *Revista Brasileira de Psiquiatria.* 2000;22:106-115.

9. Harrell F. *Regression Modeling Strategies: With Applications to Linear Models, Logistic and Ordinal Regression, and Survival Analysis.* 2nd ed. Cham, Switzerland: Springer; 2015. doi:10.1007/978-3-319-19425-7.

10. Moons KG, Donders AR, Steyerberg EW, Harrell FE. Penalized maximum likelihood estimation to directly adjust diagnostic and prognostic prediction models for overoptimism: a clinical example. *J Clin Epidemiol.* 2004;57(12):1262-1270.

11. Chaibub Neto E, Bare JC, Margolin AA. Simulation studies as designed experiments: the comparison of penalized regression models in the "large p, small n" setting. *PLoS One.* 2014;9(10):e107957.

12. Zou, H; Hastie, T. Regularization and variable selection via the elastic net.*J. R. Stat. Soc. Ser. B Stat. Methodol.* 67 (2005), no. 2, 301–320.

13. Moons KG, Altman DG, Reitsma JB, et al. Transparent Reporting of a multivariable prediction model for Individual Prognosis or Diagnosis (TRIPOD): explanation and elaboration. *Ann Intern Med.* 2015;162(1):W1-73.

14. Collins GS, Reitsma JB, Altman DG, Moons KG. Transparent Reporting of a multivariable prediction model for Individual Prognosis or Diagnosis (TRIPOD): the TRIPOD statement. *Ann Intern Med.* 2015;162(1):55-63.

15. Thapar A, Collishaw S, Pine DS, Thapar AK. Depression in adolescence. *Lancet.* 2012;379(9820):1056-1067.

16. Steyerberg EW, Vergouwe Y. Towards better clinical prediction models: seven steps for development and an ABCD for validation. *Eur Heart J.* 2014;35(29):1925-1931.

17. Austin PC, Steyerberg EW. Events per variable (EPV) and the relative performance of different strategies for estimating the out-of-sample validity of logistic regression models. *Stat Methods Med Res.* 2017;26(2):796-808.

18. Debray TP, Vergouwe Y, Koffijberg H, Nieboer D, Steyerberg EW, Moons KG. A new framework to enhance the interpretation of external validation studies of clinical prediction models. *J Clin Epidemiol.* 2015;68(3):279-289.

19. Vergouwe Y, Moons KG, Steyerberg EW. External validity of risk models: Use of benchmark values to disentangle a case-mix effect from incorrect coefficients. *Am J Epidemiol.* 2010;172(8):971-980.

20. Steyerberg EW. *Clinical prediction models: a practical approach to development, validation, and updating.* New York, NY: Springer; 2009. ​

21. Pencina MJ, Steyerberg EW, D'Agostino RB. Net reclassification index at event rate: properties and relationships. *Stat Med.* 2017;36(28):4455-4467.

22. Mari JJ, Williams P. A validity study of a psychiatric screening questionnaire (SRQ-20) in primary care in the city of Sao Paulo. *Br J Psychiatry.* 1986;148:23-26.

23. Steyerberg EW, Harrell FE. Prediction models need appropriate internal, internal-external, and external validation. *J Clin Epidemiol.* 2016;69:245-247.

24. Kessler RC, Warner CH, Ivany C, et al. Predicting suicides after psychiatric hospitalization in US Army soldiers: the Army Study To Assess Risk and rEsilience in Service members (Army STARRS). *JAMA Psychiatry.* 2015;72(1):49-57.

25. Bernardini F, Attademo L, Cleary SD, et al. Risk Prediction Models in Psychiatry: Toward a New Frontier for the Prevention of Mental Illnesses. *J Clin Psychiatry*. 2017;78(5):572-83.
